# IgG1 versus IgG3: influence of antibody-specificity and allotypic variance on virus neutralization efficacy

**DOI:** 10.3389/fimmu.2024.1490515

**Published:** 2024-10-24

**Authors:** Somanath Kallolimath, Lin Sun, Roman Palt, Esther Föderl-Höbenreich, Antonia Hermle, Leonie Voss, Marina Kleim, Falk Nimmerjahn, Johannes S. Gach, Lauren Hitchcock, Qiang Chen, Stanislav Melnik, Florian Eminger, Anja Lux, Herta Steinkellner

**Affiliations:** ^1^ Institute of Plant Biotechnology and Cell Biology, Department of Applied Genetics and Cell Biology, BOKU University, Vienna, Austria; ^2^ Diagnostic and Research Institute of Pathology, Medical University of Graz, Graz, Austria; ^3^ Division of Genetics, Department of Biology, Friedrich-Alexander-Universität Erlangen-Nürnberg, Erlangen, Germany; ^4^ FAU Profile Centre Immunomedicine, Erlangen, Germany; ^5^ Division of Infectious Diseases, University of California, Irvine, Irvine, CA, United States; ^6^ The Bio design Institute and School of Life Sciences, Arizona State University, Tempe, AZ, United States

**Keywords:** IgG3 allotypes, plant expression, SARS-CoV-2 antibodies, functional activities, antibody engineering

## Abstract

Despite the unique advantages of IgG3 over other IgG subclasses, such as mediating enhanced effector functions and increased flexibility in antigen binding due to a long hinge region, the therapeutic potential of IgG3 remains largely unexplored. This may be attributed to difficulties in recombinant expression and the reduced plasma half-life of most IgG3 allotypes. Here, we report plant expression of two SARS-CoV-2 neutralizing monoclonal antibodies (mAbs) that exhibit high (P5C3) and low (H4) antigen binding. P5C3 and H4-IgG1 mAbs were subclass-switched to IgG3 formats, designed for efficient production and increased PK values, carrying three allotypic variations, referred to as -WT, -H, and -KVH. A total of eight mAbs were produced in glycoengineered plants that synthesize fucose-free complex N-glycans with great homogeneity. Antigen, IgG-FcγR immune complex and complement binding studies demonstrated similar activities of all mAbs. In accordance, P5C3 Abs showed minor alterations in SARS-CoV-2 neutralization (NT) and antibody-dependent cell-mediated virus inhibition (ADCVI). Clear functional differences were observed between H4 variants with superior ADCVI and NT potencies of H4 IgG3 H. Our comparative study demonstrates the production of an IgG3 variant carrying an Fc domain with equivalent or enhanced functions compared to IgG3-WT, but with the stability and PK values ​​of IgG1. Our data also demonstrate that both allotypic variability and antibody specificity are important for fine-tuning of activities, an important information for the development of future therapeutics.

## Introduction

IgG3 makes up a small portion of human serum IgG and has remained relatively understudied until now. However, recent studies underscore the importance of IgG3 activities against various pathogens, especially against virus infections. While the four different IgG subclasses circulate in healthy serum in the order of IgG1 ≫ IgG2 > IgG3 > IgG4, unusually high levels of specific IgG3 Abs are detected during/post virus infection indicating a specific role to combat viral infections (1–4). In HIV vaccination programs, IgG3 has been assigned a particular role in recruiting effector function (5). Detailed studies of HIV-1-specific polyclonal serum show that IgG3 exhibits the most efficient virus neutralizing activity compared to other subtypes (6) and IgG3 antibodies seem to play a pivotal role in chikungunya virus infection (7). Interestingly, emerging evidence shows that non-neutralizing antibodies are important for immune defense through Fc-mediated effector functions, with IgG3 playing a central role (8). In accordance, recent studies show increased activity of IgG3 over IgG1 against SARS-CoV2 viruses, which is especially pronounced with mAbs that show relatively low antigen binding activities (8, 9). Further specific IgG3 related features, like the impact of the hinge region, have been reviewed recently (10–12). Although several interesting properties have been associated with IgG3, it remains unclear whether these findings are limited to specific activities or have broader applicability.

Several peculiarities distinguish IgG3 Abs from other IgG subtypes such as an elongated hinge region that provides large molecular flexibility, extensive polymorphisms, and additional glycosylation sites absent in other IgG subclasses. These characteristics make IgG3 a uniquely potent immunoglobulin, with the ability to trigger effector functions including complement activation, Ab-mediated phagocytosis (ADCP), or Ab-mediated cellular cytotoxicity (ADCC) (reviewed (11, 13)). Several studies point to activity modulation by the length of the hinge region (e.g (14).,), most probably in combination with Fc variations. Nevertheless, the existence of multiple variations, represented by the currently known 22 allotypes, complicates the elucidation of specific functional impacts. Notwithstanding, some IgG3 Fc variations are fairly well studied. For example, in contrast to other IgG subtypes which carry a histidine at the CH3 domain (H435), most IgG3 allotypes contain an arginine (R435). Consequently, these IgG3 allotypes have a shorter half-life (~ seven days) compared to other subclasses (~ 21 days) due to an impeded interaction with the neonatal Fc receptor (FcRn), responsible for IgG recycling and placental transport (15). Nevertheless, certain IgG3 allotypes carry H435 (G3m15*, G3m16*= IGHG3*17, 18,19, 22,23), which makes their PK values comparable to that of IgG1 (15). Another beneficial attribute that comes with R435 IgG3s is the purification by protein A affinity chromatography instead of protein G.

A critical factor that hampers studies on IgG3 is associated with difficulties in recombinant expression. Mammalian cell-based manufacturing is associated with low expression levels, product instability and aggregation tendencies (15), at least for the most abundant G3m5* allotype. Nevertheless, it seems that naturally occurring allotypes (G3m15*, G3m16*= IGHG3*17-19) that carry KVH mutations (CH3 domain, site 392, 397, 435) are devoid of such features as shown by G3M5* allotype (16). Despite the many differences, systematic functional studies of specific IgG3 allotypes are rare.

Here we report the systematic investigation of two well characterized SARS-CoV-2 mAb, P5C3 and H4, produced as IgG3 variants in comparison to the IgG1 orthologue. The original IgG1 subtypes with substantially different Ag-binding and virus NT properties were switched to three IgG3 variants, that carry allotypic mutations designed to overcome current limitations, i.e. low yield/quality of recombinant product and low PK values. We demonstrate the efficient expression and correct assembly of eight recombinant mAbs in engineered plants, that allow targeted N-glycosylation. The experimental setup permitted a detailed comparative study of product yield and quality. Furthermore, we demonstrate the expression of IgG3 variants with designed homogeneous N-glycosylation targeted for high effector functions. Finally, functional activities of recombinant mAbs, encompassing antigen, FcγR and complement C1q binding and virus neutralization potencies, were monitored.

## Materials and methods

### Generation of H4 and P5C3-IgG3 expression vectors

Codon-optimized (Thermofisher^®^) DNA sequences coding for heavy chain (HC) of human IgG3-WT (i.e. G3m5* allotype), as well as -KVH, and -H mutants thereof were each inserted into magnICON^®^ vector pICH26211α (**Supplementary Figure 1A**) using BsaI restriction sites (17), resulting in IgG3-WT, KVH, and -H, respectively. Codon-optimized HC variable fragment (Fv) sequences of H4 (379 bp) and P5C3 (369 bp) were grafted onto respective HC constructs resulting in H4-IgG3HC-WT/KVH/H (1512 bp) and P5C3-IgG3HC-WT/KVH/H (1503 bp). magnICON^®^ vectors carrying H4 and P5C3 kappa light chains were described recently (18–20). All constructs carry barley α-amylase signal sequence for peptide secretion. Sequence information is available in the supporting information (**Supplementary Figure 1B**.). All constructs were transformed into *Agrobacterium fabrum* (formerly *tumefaciens*) str. GV3101(pMP90), and resulting strains were used for agroinfiltration. The construction of H4 and P5C3 IgG1 vectors have been described previously (19, 20).

### In planta expression and purification of H4-, P5C3-IgG3 variants

Nicotiana benthamiana plants (ΔXTFT line (21) were grown under the conditions of 24°C, 60% humidity with a 16 h light/8 h dark photoperiod. To produce Abs, recombinant agrobacterial strains carrying the heavy chains (H4-IgG3-WT/KVH/H or P5C3-IgG3-WT/KVH/H; H4 and P5C3-IgG1) and corresponding light chains (H4-κLC or P5C3-κLC) were grown at 29°C for 24 h, centrifuged and resuspended in infiltration buffer (10 mM MES, 10 mM MgSO4; pH 5.6). Optical density of each strain was measured by extinction at 600 nm (OD600). Final OD600 of agroinfiltration mixes were set to 0.1 by dilution with infiltration buffer and delivered into leaves of 4-5 weeks old plants using a syringe.

Four days post infiltration (dpi) total soluble proteins (TSPs) were extracted from infiltrated leaves (extraction buffer: 0.5 M NaCl, 0.1 M Tris, 1 mM EDTA, 40 mM ascorbic acid; pH 7.4) in a ratio of 1:2 w/v (fresh leaf weight/buffer) for 90 min at 4°C on an orbital shaker. Subsequently, the solution was centrifuged twice at 14,000 g for 20 min at 4°C and the supernatant vacuum filtrated using 8-12 µm and 2-3 µm filters (ROTILABO^®^ Typ 12A and 15A). Recombinant IgG3-WT was purified by using protein G (Protein a, G SepharoseTM Fast Flow, GE Healthcare) and IgG3-KVH/H mutants were purified using Protein A (rProA Amicogen, Cat no: 1080025). TSP extracts were loaded at a flow rate of 1.5 mL/min on a manually packed column which was pre-equilibrated with 10 column volumes (CV) PBS (137 mM NaCl, 3 mM KCl, 10 mM Na2HPO4, 1.8 mM KH2PO4; pH 7.4). Washing was done with 20 CV PBS. Antibodies were eluted in 1 mL fractions with 0.1 M Glycine/HCl (pH 3.0), eluates were immediately neutralized with 1 M Tris (pH 9.0) and dialyzed overnight against PBS (pH 7.0).

The monomeric forms of IgG3s were separated by preparative size exclusion chromatography using HiLoad Sephadex 200/10/300 GL column (GE Healthcare). The column was equilibrated and eluted with each 1.5 CV elution buffer (PBS; 200 mM NaCl; pH 7.2) at a flow rate of 0.4 mL/min. The fractions corresponding to full length monomeric antibodies were isolated and concentrated with Amicon centrifugal filters, MWCO 10,000 kDa (Merck Millipore, UFC5010). SDS-PAGE analyses were performed in 12% gels under reducing and non-reducing conditions. Gels were stained with Coomassie Brilliant Blue R 250 staining (Carl Roth GmbH + Co. KG). Concentrations were determined by spectrophotometer (NanoDrop™ 2000, Thermo Scientific).

### N-Glycan analysis

The N-glycosylation profiles of the purified Abs were determined by mass spectrometry (MS) as described previously (9, 22). Briefly, respective heavy chains were excised from an SDS-PA-gel, digested with trypsin for all IgG3 variants, and analyzed with an LC-ESI-MS system (Thermo Orbitrap Exploris 480). The possible glycopeptides were identified as sets of peaks consisting of the peptide moiety and the attached N-glycan varying in the number of HexNAc units, hexose, deoxyhexose, and pentose residues. Manual glycopeptide searches were performed using FreeStyle 1.8 (Thermo), deconvolution was done using the extract function. The peak heights roughly reflect the molar ratios of the glycoforms. Nomenclature according to (23).

### Antigen binding ELISA

The direct sandwich ELISA was carried out as described previously (19) with modifications. In short, purified IgG1 or IgG3 mutants were coated on 96-well plates (Thermo fisher maxisorp) at concentration of 2.0 µg/mL (H4) and 0.5 µg/mL (P5C3) in PBS (pH7.4). SARS-CoV-2 spike protein RBD215 (Wuhan strain, (Shin et al., 2021)) was used as antigen in two-fold dilution with staring concentration 4.0 µg/mL and 0.5 µg/mL for H4 and P5C3 respectively. For detection, HRP-conjugated mAb CR3022 (1:15,000 in blocking buffer) was used. Absorbance was measured at 450 nm (reference 620 nm) using a Tecan Spark^®^ spectrophotometer. All samples were analyzed at least in two technical replicates. EC_50_ values were calculated by non-linear regression of the blank-corrected data points based on a four-parametric log model with GraphPad Prism (version 9).

ELISA for IgG stability testing after freeze thawing-cycles (**Supplementary Figure 3B**): 1 µg/ml RBD (100 µl per Well; Biomol) was coated in 50 mM carbonate-bicarbonate buffer pH 9.6. After incubation and washing, 200 µl of blocking buffer (PBS containing 1% bovine serum albumin) were added. Subsequently, increasing concentrations of mAb diluted (1:5) in blocking buffer were added (from 10 µg/ml to 0.64 ng/ml, 100µl per Well). Incubation steps were performed at room temperature for 1 h and followed by three washing steps with PBS/0.05% Tween-20. HRP-conjugated goat anti-human IgG Fc (Bethyl) was diluted 1:10,000 in blocking buffer. After washing, TMB solution (Invitrogen) was added to detect anti-hIgG-Fc-HRP. The reaction was stopped with 6% orthophosphoric acid (Roth). The color reaction was monitored with the SpectraMax ix3 ELISA reader (optical density (oD) at 450 nm upon 650 nm background subtraction).

### SARS-CoV-2 neutralization test

Neutralizing activity of P5C3 and H4 antibodies, was assessed by CPE assays, as described previously (24). Briefly, VeroE6 cells (VC-FTV6, Biomedica, Vienna, Austria) were seeded in 96-well plates at a density of 1x10E+4. Antibodies were serially diluted (2-fold) and incubated with SARS-CoV-2 (Delta variant GK/478K.V1 (B.1.617. 2+AY.x), GISAID name: hCoV-19/Austria/Graz-MUG21/2021) for 30 min at 37°C. Subsequently, antibody-virus mixtures were added to VeroE6 cells in sextuplicate and incubated for 96 h at 37°C. Cells were fixed with 4% neutral buffered formalin and cytopathic effects were measured by crystal violet staining. After staining, washing and drying, stain was dissolved in 10% glacial acetic acid and OD was read at 595 nm. Data were normalized to a no-antibody control (0% Inhibition) and a no-virus control (100% inhibition). IC50 values were calculated by nonlinear regression analysis with variable slopes in GraphPad PRISM Version 9.

### Immune complex preparation with microspheres

Streptavidin-coated fluorescent microspheres (Dragon Green, 200nm, Bangs Laboratories) were blocked in Blockaid solution (Invitrogen) for one hour at 50rpm and RT. Per sample, 2x1E+9 microspheres were incubated with 1.5µg biotin-labeled SARS-CoV-2 RBD (Biomol) for two hours at 50rpm RT followed by storage at 4°C overnight. The microspheres were then washed twice in Blockaid solution spinning down at 20,000xg for 3min. Subsequently, 5.0µg/ml of P5C3 or 20.0µg/ml of H4 antibody variants were added to the microspheres and incubated for two hours at 50rpm RT. Microspheres were washed with PBS by centrifugation for 3min at 20,000xg before resuspension in PBS.

### Flow cytometric assessment IC binding to primary human leukocytes

Human peripheral blood was collected from healthy donors under approval from the Ethics Committee of FAU Erlangen (Head: Kerstin Amann, Krankenhausstr. 12, D-91054 Erlangen, Germany) and upon volunteers providing their written informed consent to participate in this study.

Leucocytes were isolated by erythrocyte lysis (25). 5x1E+5 peripheral blood leukocytes (PBL) were subsequently incubated with fluorescent Dragon Green Microspheres opsonized with biotinylated RBD and P5C3 or H4 antibodies as indicated. Microspheres only coated with antigen (RBD) and PBS were used as negative controls. Cells were incubated for 2 hours on ice gently shaking before staining of surface markers to detect CD14^+^ classical monocytes, CD14^-^CD33^+^ non-classical monocytes, CD19^+^ B cells, CD56^+^CD3^-^ NK cells and CD33^+^SSC^high^ neutrophils within the living (DAPI^-^) CD45^+^ cell population upon exclusion of duplets. All antibodies were acquired from Biolegend: Brilliant Violet 510 anti-human CD14 (clone: M5E2, APC anti-human CD33 (clone: WM53), PE/Cy7 anti-human CD19 (clone: SJ25C1), PE anti-human CD3 (clone: UCHT), PE-Cyanine5 anti-human CD56 (clone: MEM-188), APC/Fire™ 750 anti-human CD45 (clone: 2D1). Data was acquired on a BD FACSCantoII and analyzed with FlowJo software. The percentage of bead-positive cells was calculated within each subpopulation. Experiments were performed with PBL from five different donors.

### Antibody-dependent cell-mediated virus inhibition assay

For the ADCVI assay 1x1E+4 VeroE6 cells per well were seeded into 96-well plates the day before the assay. The next day cells were incubated with SARS-CoV-2 WA-1/2020 (Microbiologics) at an MOI of 0.05 for 60 minutes. Unbound virus was washed off and replaced with the antibody/peripheral blood mononuclear cell (PBMC) mixture (diluted in RPMI supplemented with 1% Pen/Strep, 5% FBS). The P5C3, and H4 antibodies were serially diluted starting at 10 µg/mL and 40 µg/mL respectively. Each condition was run in duplicate. The target cell (VeroE6) to effector cell (PBMC) ratio was 1:10. PBMCs from three different healthy donors were tested. After a 72-hour incubation period at 37°C, the cells were collected, mixed with DNA/RNA shield (Zymo Research), and analyzed for nucleocapsid-specific copy numbers by quantitative PCR.

### Quantitative PCR of virus load

For qPCR, RNA was isolated from the cell lysate with a viral RNA isolation kit according to the manufacturer’s instructions (Zymo Research) and tested for nucleocapsid-specific copy numbers. Viral RNA was used in combination with 0.1 µM of each SARS-CoV-2 N-specific primer (5’-GGGGAACTTCTCCTGCTAGAAT-3’ and 5’-CAGACATTTTGCTCTCAAGCTG-3’) as well as the reagents from the qPCRBIO SyGreen 1-step Go Hi-ROX kit (PCRBIOSYSTEMS). The qPCR was carried out on an Applied Biosystems StepOne Plus real-time PCR instrument with the following program: 1 cycle at 50°C (30 min), 1 cycle at 95°C (15 min), and 45 cycles at 94°C (15 sec) and 60°C (20 sec). SARS-CoV-2 copy numbers were quantified and reported based on a standard curve generated by serial dilutions of a SARS-CoV-2 nucleocapsid-containing plasmid using the Applied Biosystems StepOne Plus real-time PCR software. Percent reduction in viral copy numbers was calculated as follows: Reduction = 1 – (copies + Ab + PBMCs)/(copies – Ab + PBMCs) x 100.

### C1q binding ELISA

Interaction of anti-RBD-IgGs with C1q was analyzed by enzyme-linked immunosorbent assay (ELISA). All incubation steps were performed at room temperature for 1h and followed by three washing steps with PBS/0.05% Tween-20. 1µg/ml of mAbs were coated in 50mM Carbonate-Bicarbonate buffer pH 9.6 (Sigma). After incubation and washing, 200µl of blocking buffer (PBS containing 3% bovine serum albumin, 0.1% gelatine and 0.05% Tween-20) were added. Afterwards, increasing concentrations of native human C1q (Sigma Aldrich) diluted in blocking buffer were added (two-fold dilutions from 65ng/µl to 2.03ng/µl) followed by incubation with HRP-conjugated sheep anti-human complement C1q (BioRad) diluted 1:500 in blocking buffer. Bound C1q was detected upon addition of TMB substrate solution (Invitrogen) and stopping the reaction with 6% orthophosphoric acid (Roth) after 5min. Thereafter, the assay was analyzed on the SpectraMax ix3 ELISA reader, where the signal intensity was measured at 450nm. Background absorbance at 650nm was subtracted.

## Results

### Production of recombinant monoclonal IgG3 variants

Previously we have established plant-based expression modules that facilitate efficient antibody subtype switch (9, 19, 20, 22, 26). In this context several monoclonal Abs (mAb) were expressed as IgG1-4 formats. While IgG 1, IgG2 and IgG4 expressed at the same range, nucleotide codon optimization was required for efficient expression of IgG3 (20). In the present study, P5C3 and H4, two broadly neutralizing anti-SARS-CoV-2 mAbs, served as a template. Both Abs are originally IgG1 Abs derived from convalescent human sera. They bind to nonoverlapping epitopes at the receptor-binding domain (RBD) of the spike protein, with significant differences in their antigen-binding potencies (19, 27, 28). While P5C3 exhibits binding affinities in the picomolar range, these values are orders of magnitude higher for H4, depending on the virus isolate (27, 28). Next to the original IgG1 format, here we generated three IgG3 variants, IgG3-WT, referring to the most prevalent human G3m5* allotype, IgG3-H (R435H), and IgG3-KVH (K392; V397; H435 present in G3m15*, 16*) (11). KVH mutations are based on previous observations on beneficial effect on IgG3 expression and purification (16) (**Supplementary Figure 1**).

**Figure 1:**
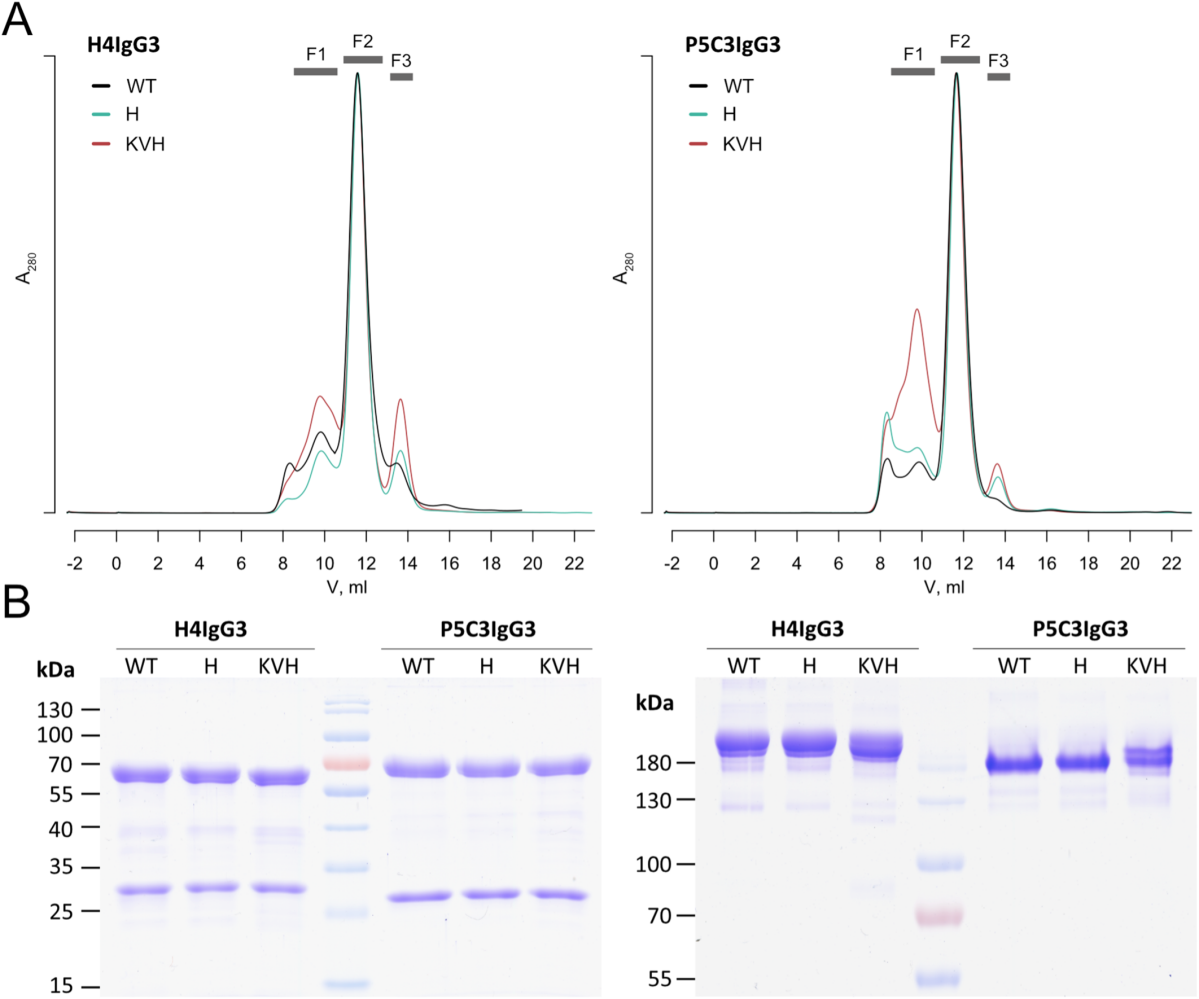
Characterization of IgG3 mAbs. **(A)** SEC profiles after Protein A purification (F1 high molecular weight, F2 fully assembled IgG3, F3 HC degradation products. **(B)** SDS PAGE from H4 and P5C3 IgG3 variants present in F2 (left: reducing, right: nonreducing). See also **Supplementary Figure 2**. Light and heavy chain (LC, HC) at position approx. 30 and 70 kDa, respectively. Fully assembled mAbs at position ~ 180 kDa. See also **Supplementary Figure 2**. Theoretical mass of heterodimeric mAbs are listed in **Supplementary Table 1**.

In total four IgG variants per antigen binding domains, H4 and P5C3, were recombinantly expressed in the glyco-engineered *Nicotiana benthamiana* line ΔXTFT (21) through agroinfiltration. Four to five days post infiltrations (dpi) IgGs were purified by affinity and subsequent size-exclusion chromatography. SEC profiles exhibited three fractions that were separately collected (F1-F3). F2, representing fully assembled mAbs, was monitored on SDS gel electrophoresis which confirmed the purity of all mAb variants (**Figure 1**). F2 was hence used for further biochemical and functional analyses. F1 and F3 represent high molecular weight Abs (most probably aggregates) and degraded HC (**Supplementary Figure 2**), respectively. H4 and P5C3 IgG1 have been characterized previously (9, 19).

While IgG3-WT carries an arginine at position 435, in KVH and H variants this amino acid was replaced by a histidine (R435H). This confers increased serum half-life and allows purification with protein A instead of protein G (15, 16). A direct comparison of purification performance using the two affinity matrices was performed with P5C3 IgG1 and IgG3-KVH (**Table 1**). The purification yield of both subtypes was slightly reduced by protein G compared to protein A. Thus, protein A was used for all purifications, except IgG3-WT where only Protein G is applicable. H4 and P5C3 IgG1 variants were expressed at yields usually obtained using the magnicon expression system (9). The purification yields of the three IgG3 variants were about 20-30% lower compared to the IgG1 orthologue (**Table 1**). Purified mAbs were stored at -20°C and stability monitored by antigen binding. All mAbs were stable at least 10 months post purification (**Supplementary Figure 3A**), however upon freeze-thawing cycles both IgG3-WT mAbs were partially degraded (**Supplementary Figure 3B**).

**Table 1:** Yield of purified mAbs. IgG3-WT was purified via protein G, other mAbs by protein A affinity.

mAbs	ProtA/G (µg/g FLW)
P5C3 IgG1	303/157
P5C3 IgG3 WT	224
P5C3 IgG3 KVH	120
P5C3 IgG3 H	190
H4 IgG1	250
H4 IgG3 WT	122
H4 IgG3 KVH	194
H4 IgG3 H	180

### Glycosylation profiles of recombinant IgG3

**Figure 2:**
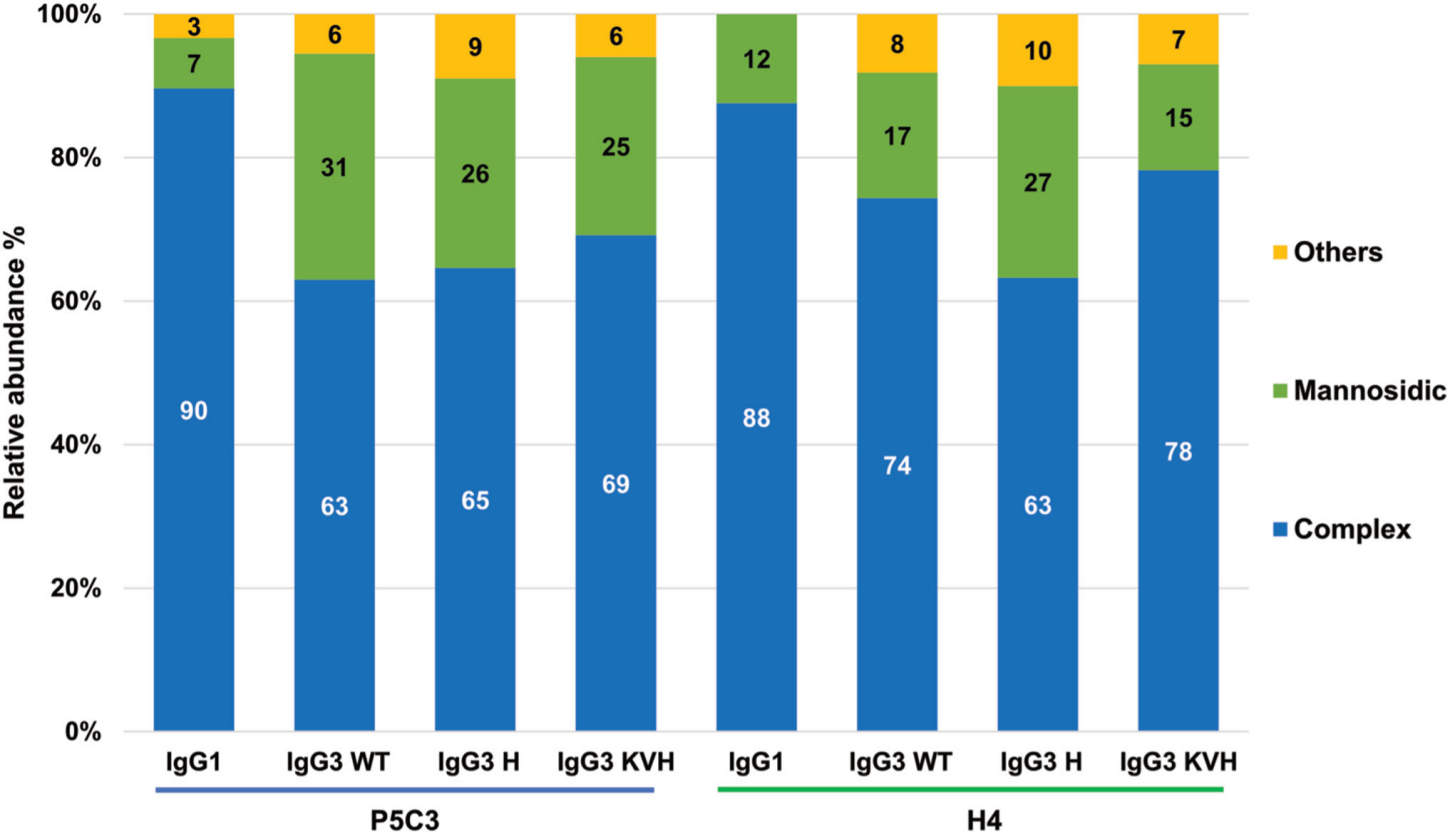
N- glycosylation profiles of purified mAbs as determined by LC-ESI-MS/MS. Bars represent the relative abundance (%) of glycoforms (for further details see **Supplementary Figure 4**). Blue bars: non-fucosylated complex GlcNAc-terminating N-glycans (mainly GnGn structures); green bars: mannosidic N-glycans (Man5-Man9). Yellow bars: combines detected glycans that occur below 3%. Nomenclature according to (23).

All IgG subtypes carry one conserved glycosite (GS) in the CH2 domain of the heavy chain at amino acid position asparagine 297 (N297). Note, IgG3 allotype G3m5* contains an additional site at position 392 in the CH3 domain, which is not present in the KVH mutant (N392K). For IgG1 multiple studies demonstrate the importance of glycosylation in immune responses (29). Here H4 and P5C3 Abs were produced in glyco-engineered *Nicotiana benthamiana* line ΔXTFT, synthesizing complex N-glycans lacking plant specific core fucose and xylose (21). To determine the N-glycosylation status of the recombinant mAbs, liquid chromatography-electrospray ionization-tandem mass spectrometry (LC-ESI-MS/MS) was performed. MS spectra of the two IgG1 formats displayed a single dominant glycoform at the Fc GS, namely xylose and core fucose-free GlcNAc-terminated structures (predominantly GnGn, 88% and 90%), accompanied by mannosidic structures (7, 12%, **Figure 2**, **Supplementary Figure 4**). Such profiles are typical for ΔXTFT-produced IgGs (21). MS spectra of IgG3 were similar but not identical compared to IgG1. While fucose/xylose free complex N-glycans are still dominating, all IgG3 variants carry increased mannosidic structures ranging from 15-31%. In the context of glycosylation, most IgG3 allotypes exhibit two peculiarities compared to other IgG subtypes, namely a potential second N-GS at CH3 domain (N392) and an O-glycosylated hinge region. We did not detect glycans at either site (**Supplementary Figure 5**). Collectively, expression profiling and biochemical analyses did not reveal obvious differences between the IgG3 variants. Importantly, all IgG3 variants carry identical glycosylation profiles. Thus, in the present study, this excludes a possible impact of this important post-translational modification on functional activities as reported previously (e.g., (12)).

### Antigen binding activities

**Figure 3:**
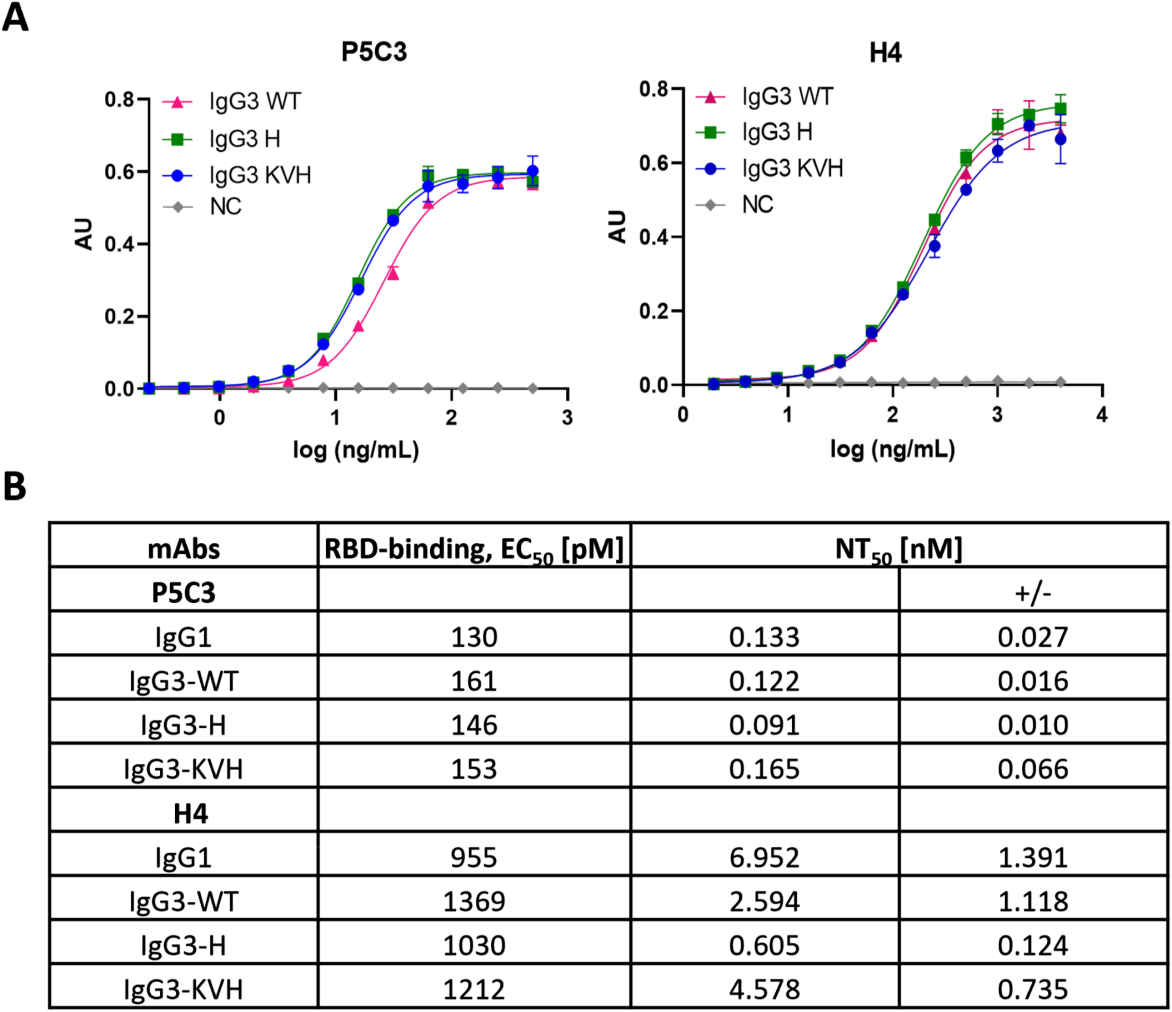
Antigen binding and neutralization activities of P5C3 and H4 mAbs. **(A)** Sandwich ELISA to determine binding activity of purified mAbs to recombinant RBD using CR3022 for detection. X-axis: concentration (ng/mL); y-axis: absorbance (AU); **(B)** Ag-binding EC_50_ and neutralization NT_50_ values. NC, negative control (irrelevant IgG1 mAb). Independent duplicates were performed.

The functional activity of anti-SARS-CoV-2 mAb variants was determined by antigen-binding assays. Direct sandwich ELISAs using SARS-CoV-2 spike protein RBD (delta variant) as antigen and HRP-labelled mAb CR3022 as secondary antibody was performed. CR3022, initially developed against SARS-CoV, broadly detects SARS-related coronaviruses (30) and does not compete with the binding of H4 and P5C3, respectively. Within mAb variants that target the identical epitope no significant differences were observed. EC_50_ values for the low binder H4 ranged between 955 and 1212 pM (**Figures 3A, B**). Accordingly, similar antigen-binding activities were observed comparing high-affinity P5C3 variants, with EC_50_ values between 130-153 pM (**Figures 3A, B**). Neither class switch nor Fc mutations had an impact on antigen-binding properties.

### Neutralization activity of IgG3 mutants

To determine the neutralization activities of P5C3 and H4 Abs, Vero cell–based SARS-CoV-2 plaque reduction assays were performed as described previously (24). All Ab variants were able to efficiently neutralize SARS-CoV-2 infection (**Figure 3B**; **Supplementary Figure 6**). However, in contrast to the antigen binding activities, clear differences in neutralization (NT) potencies were observed comparing H4 variants. All three H4-IgG3 variants had a higher NT potency compared to the IgG1 orthologue, well pronounced for IgG3-WT and -H, with 10-fold lower IC_50_ values (6.951 versus 0.559 nM). Also, different NT potencies were observed between IgG3 allelic variants, in the order of IgG3-H ≫ IgG3-WT > IgG3-KVH (IC_50_ 0.605 vs 2.594 and 4.578 nM, respectively). By contrast, no significant differences in NT activities for P5C3 variants were observed (NT IC_50_ ranging between 0.10 and 0.23 nM). Note, the remarkable high neutralization potency of P5C3 that results in especially low IC_50_ values might skew the results and activity differences between the tested variants might have been overlooked under the current settings.

### IgG immune complex binding to primary human leukocytes

In addition to virus neutralization, IgG Abs direct effector responses by binding to Fcγ receptors (FcγRs) via their Fc region. Due to the low IgG affinity of most activating FcγRs, efficient binding and subsequent FcγR activation is initiated through IgG-mediated clustering, which in turn is caused by the engagement of several antibodies on an antigen target, forming multivalent immune complex (IC). This clustering mechanism ensures that more than one IgG is present whenever effector responses occur. Recently, studies using IC and FcγR-expressing CHO cells or human peripheral blood leukocyte populations, revealed differential FcγR binding by IgG subclasses (25, 31). While FcγR-expressing cell lines are well suited to characterize specific IgG-FcγR interactions, blood leukocytes as target cells yield biologically highly relevant insights into IgG-binding in the context of appropriate receptor expression levels. Most importantly, leukocyte subsets such as CD14^+^ classical and CD14^-^ non-classical monocytes as well as neutrophilic granulocytes express multiple FcγR (activating FcγRI and activating FcγRIIa on classical monocytes, activating FcγRIIIa and FcγRIIa on non-classical monocytes, FcγRIIa/b and GPI-linked FcγRIIIb on neutrophils) that could cooperate in IC binding while NK cells and B cells only express FcγRIIIa or inhibitory FcγRIIb, respectively (32). Aiming to compare P5C3 and H4 Abs, ICs were generated using fluorescent microspheres that can be opsonized with any biotinylated antigen of choice due to loading of microspheres with streptavidin. Incubation with biotinylated SARS-CoV-2 spike RBD protein and RBD-specific P5C3 and H4 Abs thus leads to opsonization in correct conformation (33) ideal for FcγR-binding studies.

**Figure 4:**
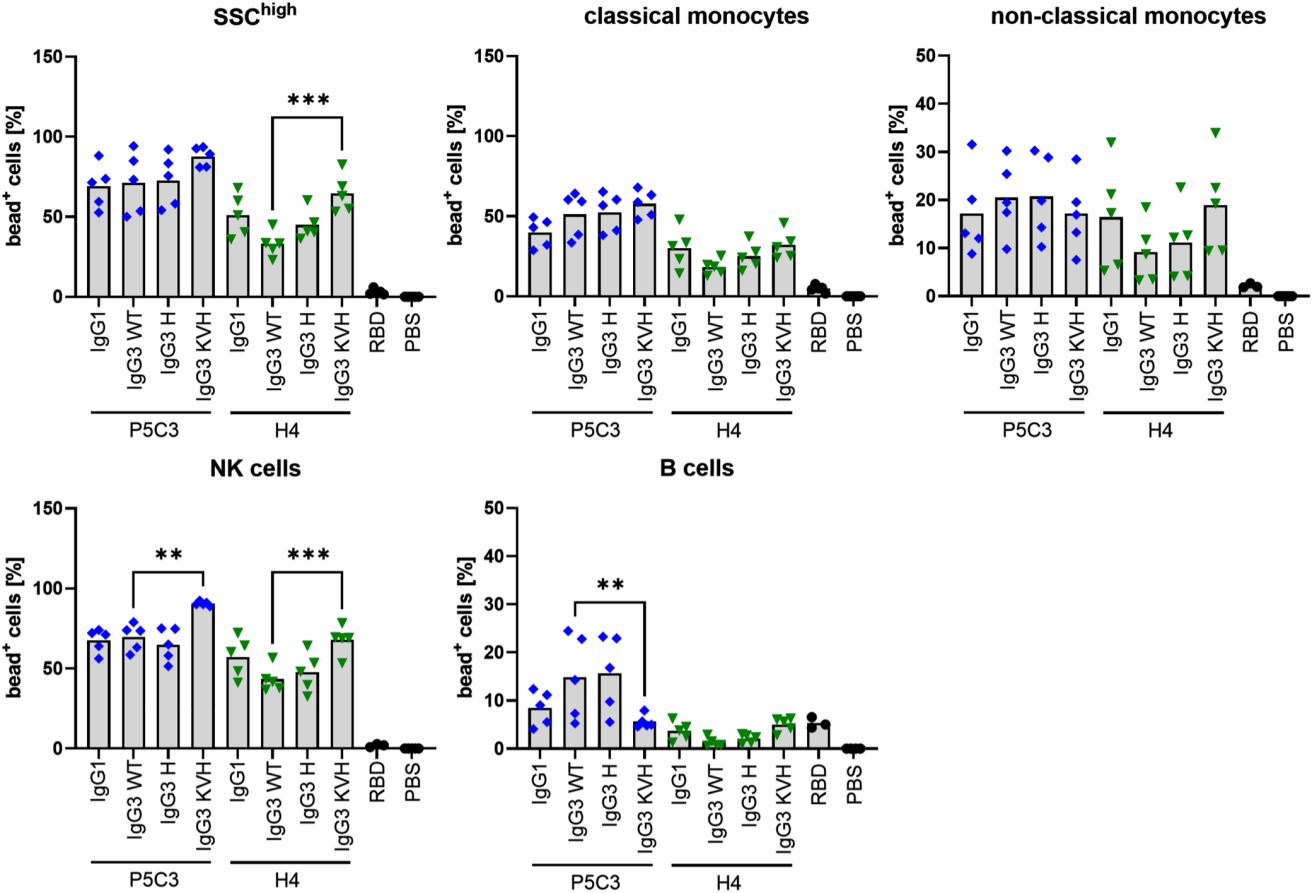
Immune complex binding to primary human leukocytes. Graphs show individual values and means of two independent experiments using a total of 5 different donors. Fluorescent microspheres were coated with RBD (or PBS as negative control) before addition of RBD-specific P5C3 or H4 mAb variants. Following incubation of microspheres with human leukocytes, flow cytometry analysis was performed and the proportion of microsphere-positive cells was calculated within the respective leukocyte populations (% bead^+^ cells on y-axis). Statistical analysis was performed by Ordinary One-way ANOVA and Holm-Sidaks multiple comparison test between IgG3 WT and IgG1, IgG3 H and KVH, respectively, following exclusion of outliers using ROUT test. P5C3 and H4-mAbs are colored in blue and green, respectively. ** p<0.01, *** p<0.001.

Generally, the results demonstrate binding of virtually all mAbs to FcγR expressing leukocytes. P5C3 mAbs generally exhibit a stronger binding compared to the H4 variants (**Figure 4**, **Supplementary Figure 4**). IC binding was most pronounced for NK cells and neutrophils both of which dominantly express FcγRIIIa/b known to interact strongly with afucosylated IgG (34). Binding to FcγRIIa/b and FcγRI expressing classical monocytes was stronger than to FcγRIIa/b and FcγRIIIa expressing non-classical monocytes and weakest for FcγRIIb expressing B cells where P5C3 IgG3-KVH and all H4 mAbs were comparable to the RBD control. Importantly, compared to IgG3-WT and the IgG1 orthologue, both IgG3-H variants displayed similar binding behavior to all cell types suggesting functional equivalence of this variant. In contrast, binding of KVH mAbs was found to be notably increased or decreased in a cell-type dependent manner indicating altered binding to especially to FcγRIIIa/b and FcγRIIb.

### Antibody-dependent cell-mediated virus inhibition

**Figure 5:**
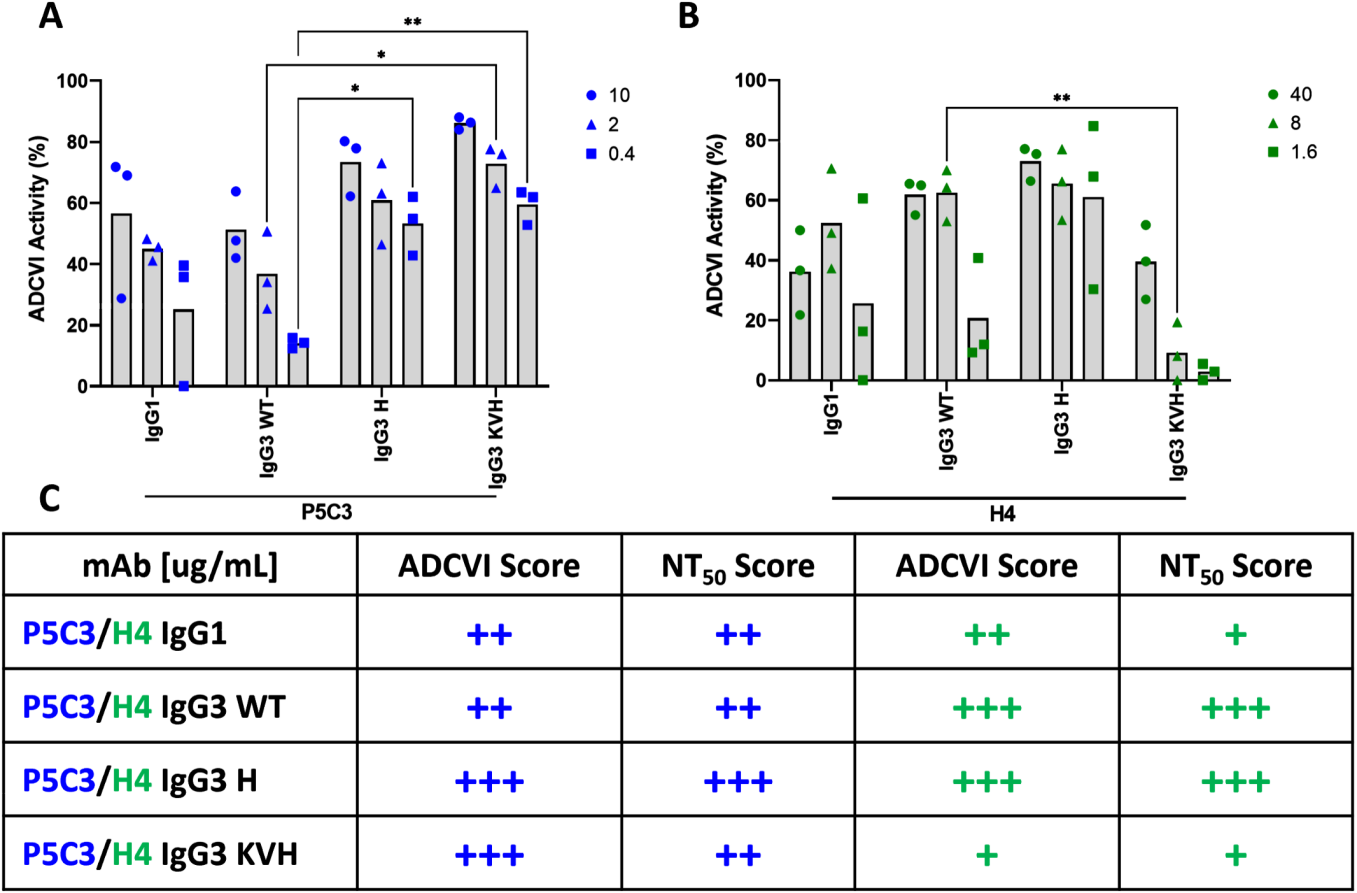
ADCVI activity assay. Each antibody variant was tested for its ability to induce effector functions. The average ADCVI activity for the P5C3 **(A)** and H4 mAb panels **(B)** tested at three different concentrations is shown on the y-axis. Numbering on the right side refers to Ab concentration in µg/mL. Bars represent the mean of three independent experiments with different PBMC donors (n=3). Statistical analysis was performed by 2way ANOVA and Dunnett’s multiple comparisons test between IgG3 WT and IgG1, IgG3 H, and IgG KVH, respectively (GraphPadPrism 10.2). * p<0.05, ** p<0.01; **(C)** Comparative scoring of ADCVI data and NT_50_ values within P5C3 and H4 Abs. + low, ++ medium, +++ high activity.

We further investigated antibody-mediated effector functions of the P5C3 and H4 Abs in the presence of human effector cells (PBMCs) and SARS-CoV-2 infected target cells (VeroE6) using an ADCVI assay. ADCVI is a measure of FcγR-mediated antiviral activity and occurs when antibodies bound to virus-infected target cells engage FcγR-bearing effector cells. The resulting virus inhibition is due in part to target cell death mediated through ADCC and/or ADCP and in part to non-cytolytic mechanisms such as β-chemokine release from the effector cells. ADCVI activity has been particularly observed with protection from lentivirus infections (35, 36). In this study we assessed the ADCVI activity of the P53C and H4 variants at three mAb concentrations. For P5C3 mAbs we detected a similar ADCVI activity trend: KVH > H ≥ IgG1 > WT in all three antibody concentrations tested (i.e., 10, 2, and 0.4 µg/mL) (**Figure 5A**). Interestingly, ADCVI was more pronounced in the H4 Abs (Ab concentration: 40, 8, and 1.6 µg/mL): H≥ WT ≥ IgG1 ≫KVH. (**Figure 5B**). Overall, ADCVI levels decreased in a dose-depended manner, however the ADCVI activity for H4 IgG3 H retained higher activities compared to the other variants even at a concentration of 1.6 µg/mL.

### Complement C1q binding

**Figure 6:**
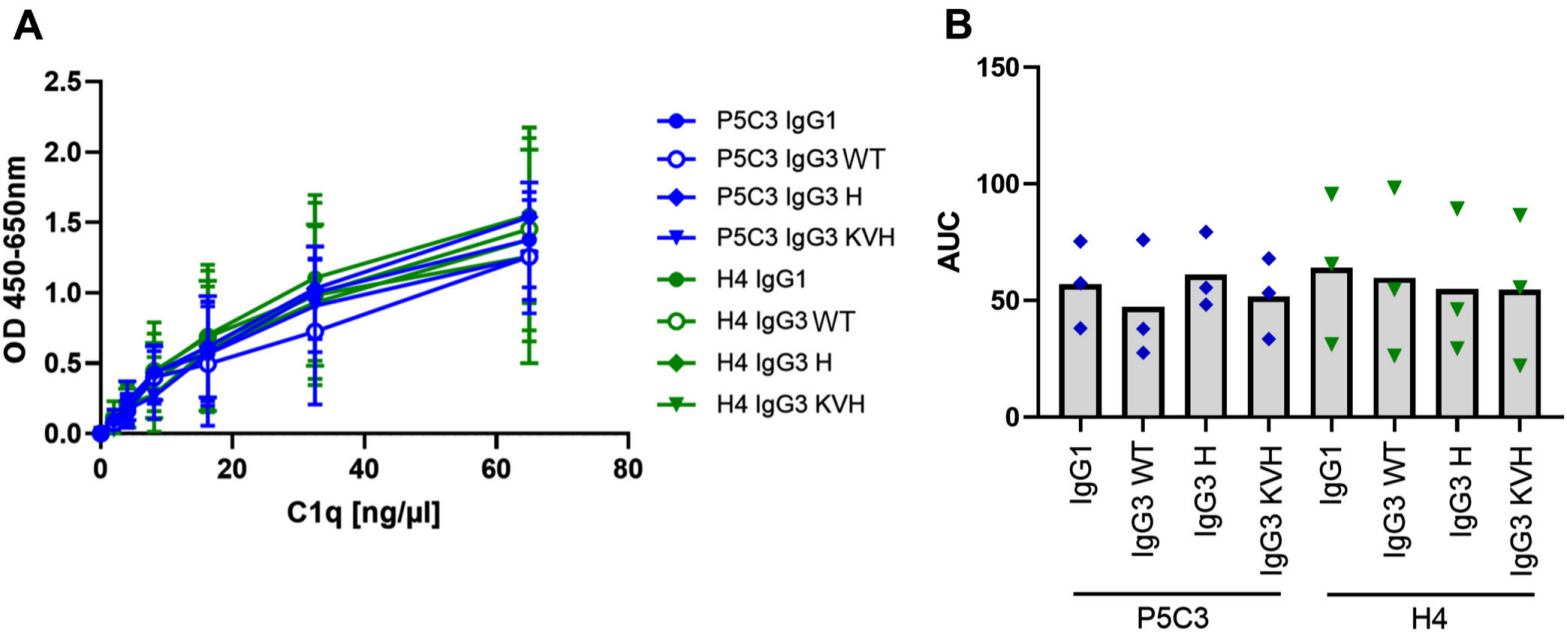
Complement C1q binding. The capacity to recruit human C1q was compared for P5C3 and H4 mAbs. **(A)** mAbs were coated and recombinant human C1q was added in increasing concentrations. Mean and standard deviation is shown for three independent experiments performed in technical duplicates. **(B)** AUC was extracted, and statistical analysis was performed by Friedman test and Dunn´s multiple comparisons test between IgG3 WT and IgG1, IgG3 H and KVH, respectively.

Beyond induction of FcγR-dependent effector functions, IgG3 is also a prominent activator of the complement cascade (8, 37). Activation of the classical complement pathway occurs when IgG is deposited on a target cell surface leading to recruitment of C1q. Binding of C1q initiates the C1 serin protease activity resulting in sequential cleavage of downstream complement proteins. Ultimately, complement activation results in formation of membrane attack complexes directly lysing the target cell. Alternatively, opsonization with complement fragments may induce phagocytic uptake via complement receptors expressed on myeloid effector cells (38). As activation of the complement system was also shown to be relevant for anti-viral immune responses (39) we further compared C1q binding (**Figure 6**). Overall, all P5C3 and H4 IgG1 as well as IgG3 mAb variants were able to interact with C1q comparably confirming their functional equality for this effector function.

## Discussion

Here we evaluated the impact of three anti-SARS-CoV2 IgG3 allotypic variations in terms of product yield and quality in comparison to their respective IgG1 orthologues. Regarding the production process, we do not observe obvious differences in the expression levels between the three IgG3 variants. Moreover, IgG3 expresses at similar levels to IgG1, which came as a surprise, since previous plant-based production resulted in at least 10 times lower expression levels of IgG3 compared to the other IgG subtypes (9, 26). Efficient expression may be due to the new gene codon optimization strategies used in this study and recently evaluated (20). Another observation that came as a surprise was the potential aggregation of IgG3 post protein A purification. In the context of mammalian cell production IgG3-WT showed substantial aggregate formation which could be reduced by KVH mutation (16). We do not observe this in the plant-based production, KVH exhibits slightly more high molecular weight forms post protein A purification than IgG3-WT and -H. Regarding stability we do not see any difference between IgG3 and IgG1 within 6-10 months of storage at -20°C in PBS. However, a systematic evaluation including repeated thawing cycles revealed quality loss of IgG3-WT, while all other Abs remained stable. This indicates the improved quality of the IgG3-KVH and -H variants. Note that no detailed optimization in storage conditions, like different buffer compositions, were performed.

Despite accounting for only 2-3% of the mass of an IgG molecule, glycans affect critical Ab functions such as Fc receptor binding and subsequent effector functions and serum half-live, which is particularly well known for IgG1 (29). Here we show virtually identical glycosylation profiles for all recombinant mAbs. CH3 amino acid differences did not impact glycan composition, consistent with the observation that serum-derived IgG1 and IgG3 carry a similar N-glycosylation pattern (40). Importantly, we produced mAbs with a largely homogeneous N-glycosylation profile, namely fucose-free GnGn structures Glycan homogeneity not only meets high production standards, but also facilitates further glycan engineering processes (e.g (41, 42).,).

Although frequently discussed so far there is no clear data on the occupancy of the second potential GS at the CH3 domain (N392). Our studies did not reveal glycans at that site. Some reports point to the importance of N392 for IgG3 stability (43).

A peculiarity that IgG3 shares with IgA1 Abs is an extended O-glycosylated hinge, often associated with enhanced proteolytic degradation (44, 45). Approximately 10% of human serum IgG3 contains O-linked glycans, even less in mammalian cell-derived monoclonals (44). None of the plant-produced IgG3 variants in this study carried O-glycans in IgG3 hinge repeats.

While a series of studies show increased IgG3 effector functions compared to IgG1, less is known about how this IgG subclass can affect binding and neutralization of viruses (10, 11, 13). We found no significant differences in antigen binding upon subtype and allotype switch, in line with previous observations (9, 19, 22, 46, 47). However, that does not seem to be a general rule since several lines of evidence document the contribution of the Ab constant domain to the manifestation of variable region and their interaction with antigens (46, 48).

Notably, we observe Ab-specific NT activity. While all P5C3 variants (RBD affinity in pM range) show similar SARS-CoV2 NT, irrespective of their Fc domain, this activity differs between H4 Abs (RBD affinity in μM range). H4 IgG1 and IgG3-KVH exhibit an approx. 10 times lower NT compared to IgG-WT and -H. Our results corroborate a recent study that report on influenza virus and SARS-CoV-2 mAbs (46). When affinity for antigen is high, the NT differences are minor, but as the affinity for antigen is reduced through antigenic variation, it was found that antibody constant domains can significantly alter binding and neutralization capacity (46).

The decreased NT_50_ value of H4 IgG3-KVH to the level of H4 IgG1 might be associated with the lysine at position 392, also present in IgG1. By contrast, most IgG3 allotypes, including H4 IgG3-WT and -H, carry an asparagine at this site. A structural impact of K/N392 on IgGs has been suggested previously, with so far unclear functional consequences (43). Notwithstanding, a recent study reports increased binding to some Fcγ-receptors and as a consequence enhanced ADCC activity of IgG3-N392 compared to K392 allotypes (49). H4 IgG3-H exhibited identical potent NT compared to IgG3-WT. From a therapeutic point of view the H variant is particularly interesting, since it overcomes current limitations (low expression, instability) but carries the potential IgG3-associated advantages (high effector function and PK values).

Mounting evidence points to the importance of the interplay of Ab NT and Fc-mediated activity in SARS-CoV-2 (50–52) and other viral infections e.g. HIV (53–55). We thus determined immune cell binding to IgG in the form multivalent IC closely resembling IgG opsonized viral particles. Albeit we observed a tendency of enhanced binding of P5C3 IgG3-WT (but not H4 IgG3-WT) to B cells and non-classical monocytes in comparison to the respective IgG1 orthologues, no significant differences could be detected. This is contrary to several previous studies demonstrating an increase in FcγR binding of IgG3 over IgG1 (31, 49, 56) and can potentially be attributed to the IgG1 and IgG3-WT allotypes used in this study (G1m17,1 and G3m5*). While it is now increasingly clear that allotypes have a significant impact on FcγR binding (12, 49) this might have been overlooked in the past. In any case, previous studies either did not indicate which IgG allotypes were used (56) or experiments were conducted comparing other allotype combinations (8, 25, 31, 49). An additional confounding factor could be the experimental setup to assess FcγR and/or immune cell binding employing monomeric IgG and FcγR for interaction analysis by surface plasmon resonance (SPR) (12, 49, 56), different forms of IgG immune complex generation most likely resulting in ICs of varying multivalency (12, 25, 31, 56) or functional readouts e.g. phagocytic uptake or ADCC induction (8, 12, 49). In comparison to IgG3-WT, the R435H replacement in IgG3-H did not alter immune cell binding. More pronounced interaction with neutrophils and NK cells but comparable or even reduced binding to monocytes and B cells, respectively, was however seen for the KVH variant. G3m15* (IGHG*17) which naturally contains KVH was previously described to have increased or decreased Fc effector functions depending on the antigen specificity of the investigated mAbs despite no major changes in FcγR affinity as determined by SPR (12, 49). Possibly, immune cell binding observed for IgG3-KVH ICs is a consequence of KVH introduced on the G3m5* backbone thus generating a novel mAb variant not previously studied but with the potential of increased cytotoxic activity.

We additionally investigated interaction of P5C3 and H4 mAbs with C1q as an indicator of their capacity to activate the complement system. In accordance with previous studies showing comparable complement activation by IgG3 allotypes (37, 49), no differences between IgG3 variants could be observed.

Ultimately, and in line with antigen and immune cell binding, we found little variation of ADCVI activity between the IgG1 and IgG3-WT variants. However, in both P5C3 and H4, the H variant shows a slightly increased virus inhibition that might be explained by lower EC_50_ and NT_50_ in comparison to IgG3-WT given that efficient antigen binding as well as effector cell activation are prerequisites for ADCVI activity. This further demonstrates the significance of the H variant. The dramatic loss of ADCVI of H4-KVH (especially at low concentrations) might be associated with the presence of N392L which can lead to decreased ADCC activity as previously reported (49).

Currently, we cannot provide a complete explanation of the molecular mechanisms responsible for the observed functional differences. Nevertheless, the fact that H4 IgG3 H outperforms the other variants in both NT and ADCVI (**Figure 5C**) makes this Ab variant especially interesting in the context of low- affinity Abs. Although both activities are mechanistically different, cross-linking activities are clearly taking place. Functional differences between IgG3 and other subclasses are often attributed to the altered hinge region in IgG3 (e.g (14, 57). However, we can definitively rule this out as all IgG3 mAbs carry the identical hinge domains. It is noteworthy that 22 IgG3 allotypes are currently known (11) and publications usually refer to only one of them., i.e. G3m5*. However, each allotype carries a specific Fc sequence which might alter Ab properties. Future studies that expand beyond the functions examined here, to capture a broader range of antibody antiviral activities across a dynamic range of antibody concentrations, may provide further insights into the complex relationship between Fc- and Fab- activities, and more importantly, how these may be harnessed by next generation of therapeutics and vaccines. IgG3 variants that carry 435H have a half-live equivalent to IgG1 and there is no evidence that allelic mismatch causes any clinical adverse effects (reviewed (11)). Therefore, next-generation therapeutic antibodies should consider utilizing IgG3 backbones due to the greater molecular flexibility and potential high-effector functions.

## Data Availability

The datasets presented in this study can be found in online repositories. The names of the repository/repositories and accession number(s) can be found below: GPST000472 (GycoPost, PIN: 3075).
