# Supplementary material for: IgG1 versus IgG3: influence of antibody-specificity and allotypic variance on virus neutralization efficacy
Source: Front Immunol. 2024 Oct 24;15:1490515. doi: 10.3389/fimmu.2024.1490515 (PMC11540624; doi:10.3389/fimmu.2024.1490515)
Supplement: Supplementary file 1 [file DataSheet1.pdf]

Supplementary Materials

A

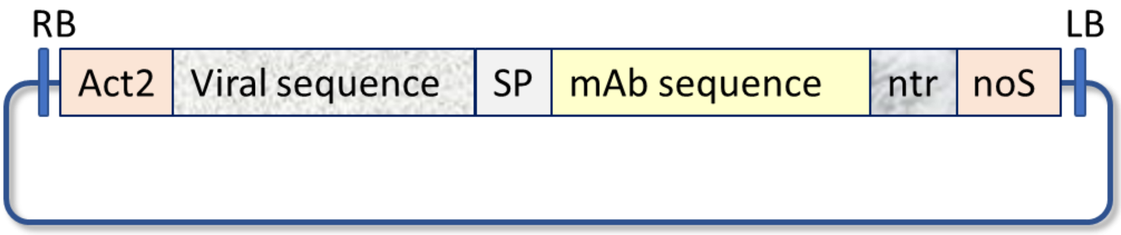

pICH26211α-mAb

B

IgG1Hc (IGHG1*01 or G1m17,1)

ASTKGPSVFPLAPSSKSTSGGTAALGCLVKDYFPEPVTVSWNSGALTSGVHTFPAVLQSSGLYSLSSVVTVPSSSLGT
QTYICNVNHKPSNTKVDKKAEPKSCDKTHTCPPCPAPELLGGPSVFLFPPKPKDTLMISRTPEVTCVVVDVSHEDPE
VKFNWYVDGVEVHNAKTKPREEQYNSTYRVVSVLTVLHQDWLNGKEYKCKVSNKALPAPIEKTISKAKGQPREPQ
VYTLPPSRDELTKNQVSLTCLVKGFYPSDIAVEWESNGQPENNYKTTPPVLDSDGSFFLYSKLTVDKSRWQQGNVFS
CSVMHEALHNHYTQKSLSLSPGK

IgG3Hc_WT (IGHG3*01 or G3m5*)

ASTKGPSVFPLAPCSRSTSGGTAALGCLVKDYFPEPVTVSWNSGALTSGVHTFPAVLQSSGLYSLSSVVTVPSSSLGT
QTYTCNVNHKPSNTKVDKRV**ELKTPLGDTTHTCPRCPEPKSCDTPPPCPRCPEPKSCDTPPPCPRCPEPKSCDTPP**
PCPRCPAPELLGGPSVFLFPPKPKDTLMISRTPEVTCVVVDVSHEDPEVQFKWYVDGVEVHNAKTKPREEQFNSTF
RVVSVLTVLHQDWLNGKEYKCKVSNKALPAPIEKTISKTKGQPREPQVYTLPPSREEMTKNQVSLTCLVKGFYPSDIA
VEWESSGQPENNY**NTT**PPMLDSDGSFFLYSKLTVDKSRWQQGNIFSCSVMHEALHNRFTQKSLSLSPGK

IgG3Hc_KVH

ASTKGPSVFPLAPCSRSTSGGTAALGCLVKDYFPEPVTVSWNSGALTSGVHTFPAVLQSSGLYSLSSVVTVPSSSLGT
QTYTCNVNHKPSNTKVDKRV**ELKTPLGDTTHTCPRCPEPKSCDTPPPCPRCPEPKSCDTPPPCPRCPEPKSCDTPP**
PCPRCPAPELLGGPSVFLFPPKPKDTLMISRTPEVTCVVVDVSHEDPEVQFKWYVDGVEVHNAKTKPREEQFNSTF
RVVSVLTVLHQDWLNGKEYKCKVSNKALPAPIEKTISKTKGQPREPQVYTLPPSREEMTKNQVSLTCLVKGFYPSDIA
VEWESSGQPENNY**K**TTPP**V**LDSDGSFFLYSKLTVDKSRWQQGNIFSCSVMHEALHN**H**FTQKSLSLSPGK

IgG3Hc_H

ASTKGPSVFPLAPCSRSTSGGTAALGCLVKDYFPEPVTVSWNSGALTSGVHTFPAVLQSSGLYSLSSVVTVPSSSLGT
QTYTCNVNHKPSNTKVDKRV**ELKTPLGDTTHTCPRCPEPKSCDTPPPCPRCPEPKSCDTPPPCPRCPEPKSCDTPP**
PCPRCPAPELLGGPSVFLFPPKPKDTLMISRTPEVTCVVVDVSHEDPEVQFKWYVDGVEVHNAKTKPREEQFNSTF
RVVSVLTVLHQDWLNGKEYKCKVSNKALPAPIEKTISKTKGQPREPQVYTLPPSREEMTKNQVSLTCLVKGFYPSDIA
VEWESSGQPENNY**NTT**PPMLDSDGSFFLYSKLTVDKSRWQQGNIFSCSVMHEALHN**H**FTQKSLSLSPGK

κLc

RTVAAPSVFIFPPSDEQLKSGTASVVCLLNNFYPREAKVQWKVDNALQSGNSQESVTEQDSKDSTYSLSSTLTLSKA
DYEKHKVYACEVTHQGLSSPVTKSFNRGEC

H4_HcFv

QVQLVQSGAEVKKPGASVKVSCKASGYTFTGYYMHWVRQAPGQGLEWMGRINPNSGGTNYAQKFQGRVTMT RDTSISTAYMELSRLRSDDTAVYYCARVPYCSSTSCHRDWYFDLWGRGTLVTVSS

H4_LcFv

DIQMTQSPLSLPVTPGEPASISCRSSQSLLDSDDGNTYLDWYLQKPGQSPQLLIYTLSYRASGVPDRFSGSGSGTDFT LKISRVEAEDVGVYYCMQRIEFPLTFGGGTKVEIK

P5C3_HcFv

QMQLVQSGPEVKKPGTSVKVSCKASGFTFTSSAVQWVRQARGQRLEWIGWIVVGSGNTDYAQQFQERVTITRD MSTSTAYMELSSLGSEDTAVYYCAAPNCSGGSCYDGFDLWGQGTMVTVSS

P5C3_LcFv

EIVLTQSPGTLSLSPGERATLSCRGSQSVRSSYLGWYQQKPGQAPRLLIYGASSRATGIPDRFSGSGSGTDFTLTISRLE PEDFAVYYCQQYGSSPWTFGQGTKVEIK

S Figure 1: A: schematic presentation of the expression vector pICH26211α-mAb. Act2: actin 2 promoter; viral sequence: TMV 5` domain; SP: α-amylase signal peptide; ntr: TMV 3`nontranslated region; noS: nos terminator; RB, LB: right and left border; **B:** Amino acid sequence of IgG1 and IgG3 heavy chain (Hc) allotypes; H4 and P5C3 variable heavy and light chains (HcFv and LcFv), respectively. Red underlined: conserved Fc N-glycosylation site; red bold: amino acid differences

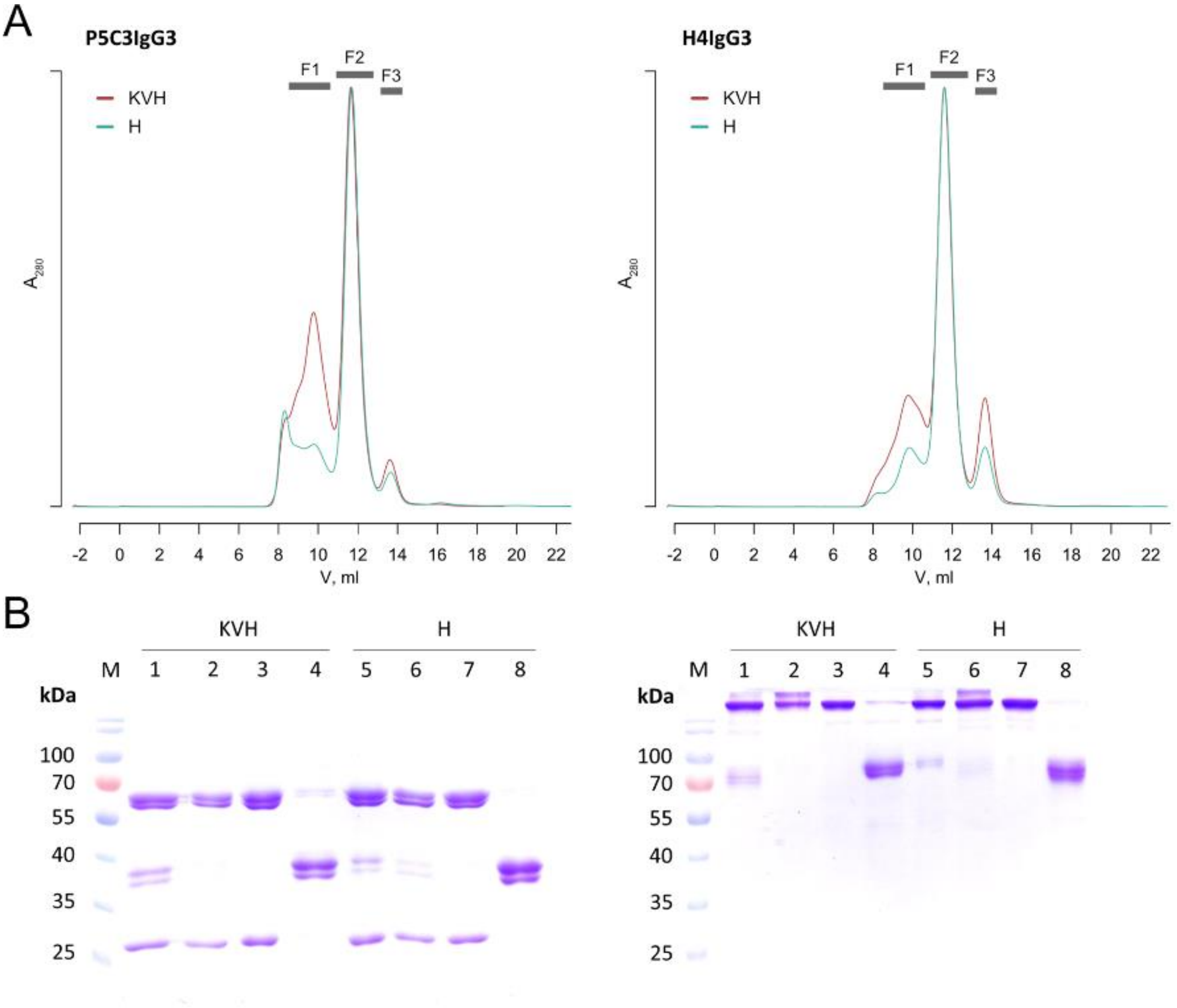

S Figure 2: A: SEC profiles after Protein A purification of IgG3 mAbs; **B:** SDS PAGE from H4IgG3 (left: reducing, right: nonreducing). Line 1: Protein A purified IgG3 (represent all fractions seen in the SEC profile); line 2-4: samples collected from fraction F1 – F3. For functional assays mAbs recruited from F2 were used.

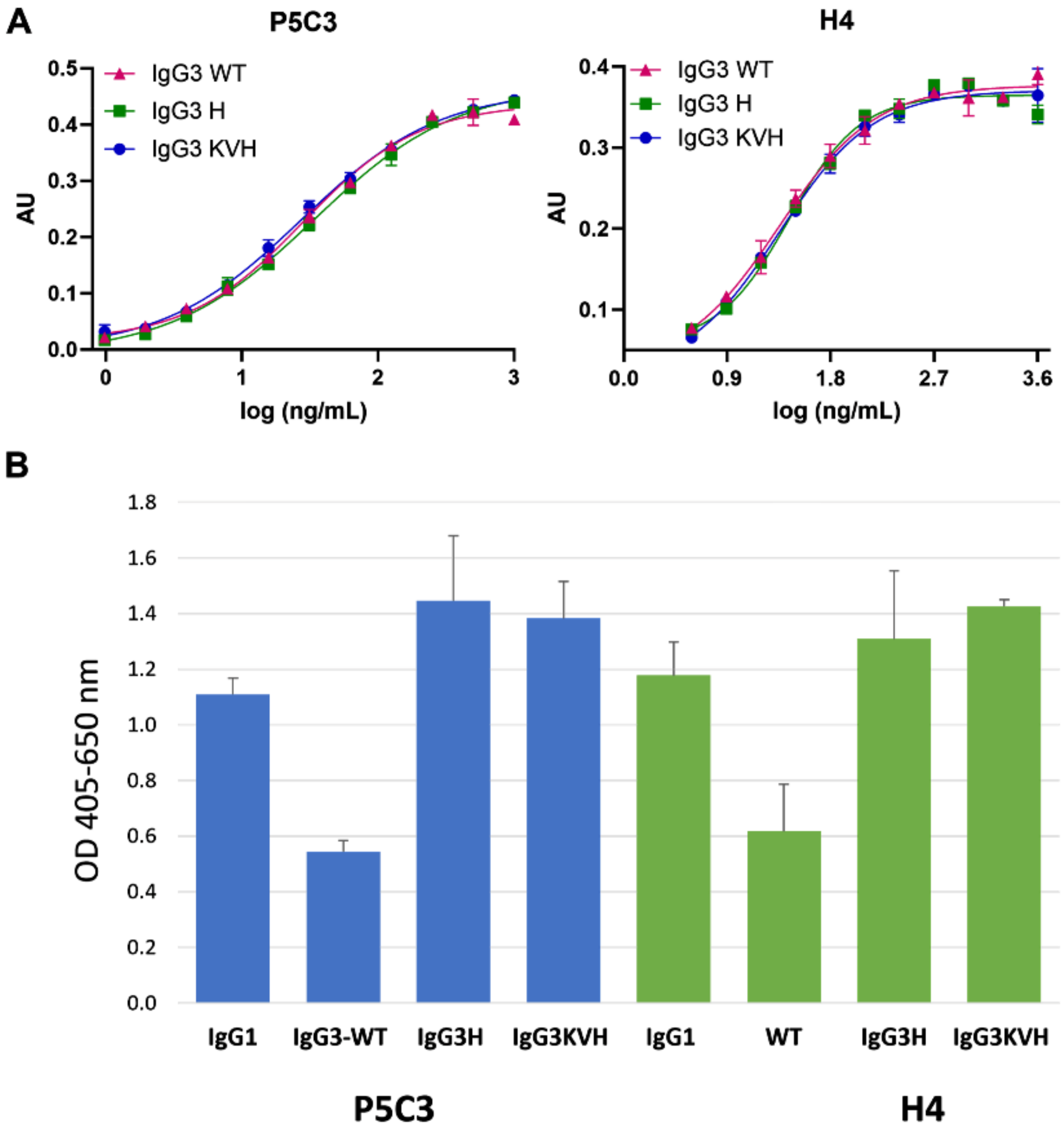

S Figure 3: A: Long term (7-10 months) freezed P5C3/H4IgG3 mAbs stability test by using RBD binding ELISA assay (further information see materials and methods).; **B:** IgG stability control after several freeze-thaw cycles (binding activity is shown in bars, further information see materials and methods).

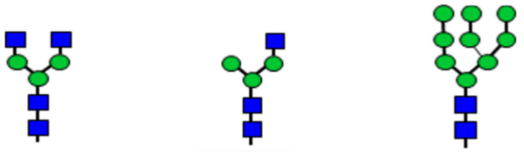

mAbs	GnGn	GnM	Mannosidic (Man7-9)	Others (>1,5 %)
**P5C3IgG WT**	55.1	9.6	34.1	1.2
**P5C3IgG3 H**	59.2	8.2	31.0	1.5
**P5C3IgG3 KVH**	61.4	8.9	28.5	1.2
**H4IgG2 WT**	70.7	10.7	16.2	2.3
**H4IgG3 H**	57.7	8.4	31.4	2.5
**H4IgG3 KVH**	70.6	10.2	17.3	1.8

S Figure 4: The relative abundance of glycoforms (%) present at IgG3 Fc-GS. Others =∑of glycoforms less than 1,5%. Glycan nomenclature according to Altmann et al. (2024).

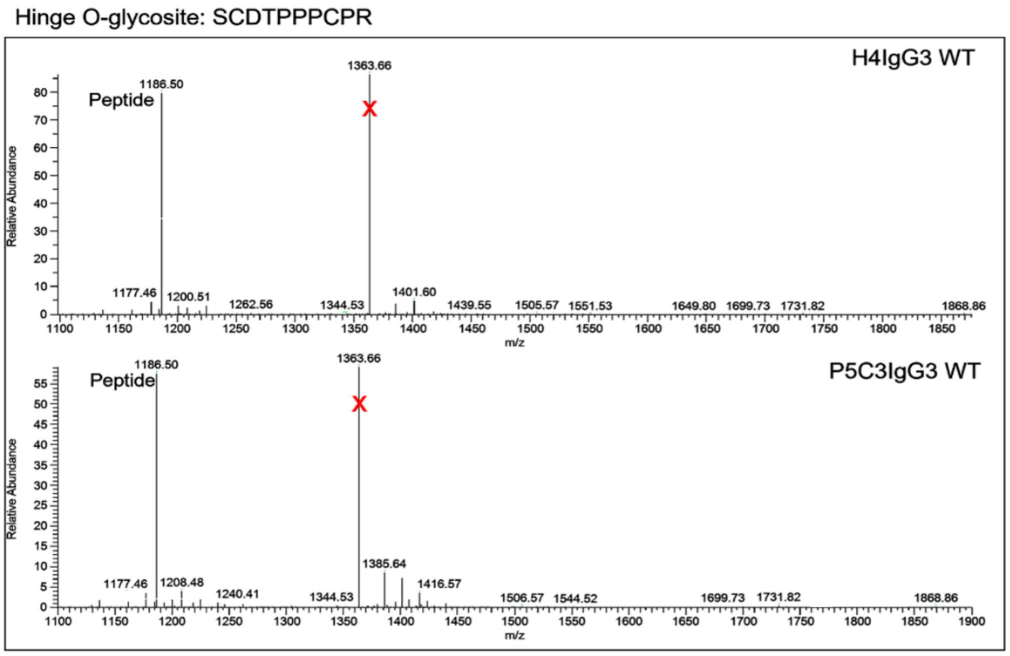

S Figure 5: Evaluation of hinge O-glycosylation. LC-ESI-MS profile of the hinge region peptide (SCDTPPPCPR, 1186.50 [M+H]), peak marked with “x” at 1363.66 refers to a co-eluted peptide.

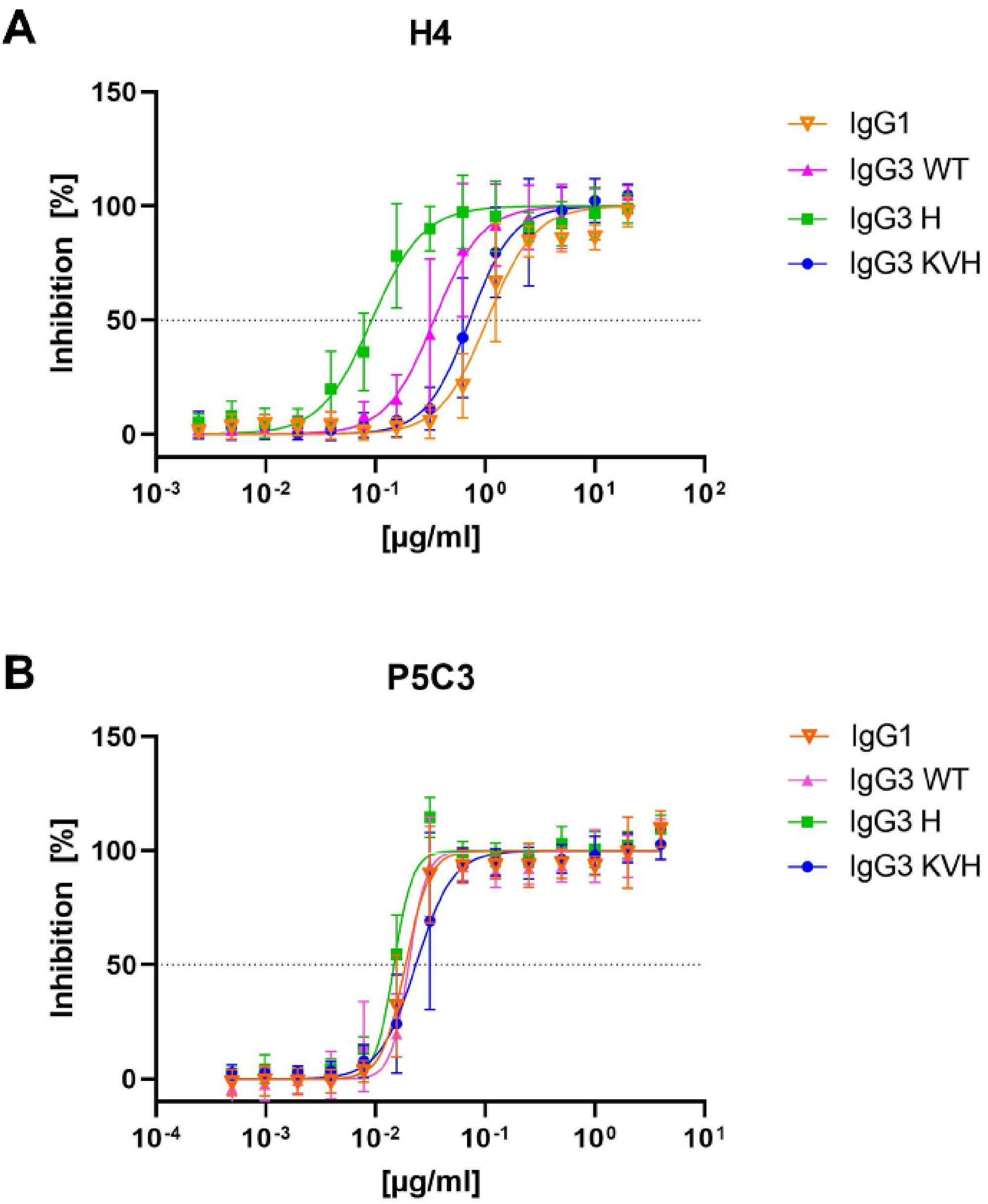

S Figure 6: Dose response curve of monoclonal antibodies against authentic SARS-CoV-2 in VeroE6 cells. Virus was preincubated with antibodies prior to infection. Cell monolayers were at MOI = 0.01and incubated for 96 h. Cytopathic effects were assessed by crystal violet staining. Infected, untreated cells were set to 0 % inhibition and uninfected, untreated cells to 100 % inhibition and IC50 values were calculated by non-linear regression analysis. Values are sextuplicate from two independent experiments each conducted in sextuplicate and are presented as mean +/- SD.

S Table 1: Molecular mass of each antibody

**Ab type**	**Mol. mass (kDa) of assembled heterodimer**
P5C3IgG1	150.740
P5C3IgG3 WT	161.060
P5C3IgG3 KVH	161.060
P5C3IgG3 H	161.060
H4IgG1	151.040
H4IgG3 WT	161.380
H4IgG3 KVH	161.380
H4IgG3 H	161.380
